# Whey protein isolate inhibits hepatic FGF21 production, which precedes weight gain, hyperinsulinemia and hyperglycemia in mice fed a high-fat diet

**DOI:** 10.1038/s41598-020-72975-8

**Published:** 2020-09-25

**Authors:** Katsunori Nonogaki, Takao Kaji

**Affiliations:** Laboratory of Diabetes and Nutrition, Tohoku University New Industry Creation Hatcher Center, 6-6-11 Aramakiaza-Aoba, Aoba-ku, Sendai, Miyagi 980-8579 Japan

**Keywords:** Physiology, Endocrinology, Medical research

## Abstract

Insufficient expression of hepatic fibroblast growth factor 21 (FGF21) and stromal cell-derived factor 2 like 1 (Sdf2l1) reportedly leads to insulin resistance and hepatosteatosis in obesity and type 2 diabetes. On the other hand, increased expression of hepatic serotonin receptor 2a (htr2a) in diet-induced obesity contributes to hepatosteatosis. Here we show that increases in circulating FGF21 levels and expression of hepatic FGF21 preceded weight gain, hyperinsulinemia, and hyperglycemia in C57BLJ6 mice fed a high-fat diet. Expression of hepatic htr2a and Sdf2l1 increased in insulin-resistant mice fed a high-fat diet. Intake of whey protein isolate decreased plasma FGF21 levels and expression of hepatic FGF21 in mice fed either a high-fat diet or a chow diet, whereas it only suppressed the overexpression of hepatic Sdf2 and htr2a in insulin-resistant mice fed a high-fat diet. Moreover, intake of whey protein isolate decreased plasma serotonin levels in mice fed either a high-fat diet or a chow diet. Genetic inhibition of tryptophan hydroxylase 1 decreased hepatic FGF21 expression and plasma FGF21 levels in mice. These findings suggest that increased hepatic FGF21 production precedes diet-induced weight gain, hyperinsulinemia, and hyperglycemia, and that intake of whey protein isolate could inhibit hepatic FGF21 production by suppressing peripheral serotonin synthesis.

## Introduction

Although lifestyle control, including a healthy diet, physical activity, and maintaining a normal body weight, is suggested to prevent the onset of type 2 diabetes, the prevalence of diabetes continues to increase worldwide. Insulin resistance is a primary metabolic disorder and a target for the prevention of type 2 diabetes. Recent studies suggest that altered expression of genes in the liver following feeding substantially contributes to insulin resistance in the development of obesity and type 2 diabetes^1–10^.

Fibroblast growth factor 21 (FGF21) is widely expressed in various organs, including the liver, pancreas, skeletal muscle, and adipose tissues, but circulating FGF21 in diet-induced obesity and type 2 diabetes is liver-derived^1^. Although FGF21 has several beneficial effects for glucose and lipid metabolism^1,2^, circulating FGF21 levels are paradoxically increased in hepatosteatosis, obesity, and type 2 diabetes^1–8^. Mice with liver-specific FGF21 knockout fed a high-fat diet exhibit enhanced insulin resistance, suggesting that higher plasma FGF21 levels represent a compensatory response to metabolic disturbances in obesity and/or type 2 diabetes^1^.

In addition, termination of endoplasmic reticulum (ER) stress responses in the liver following feeding by stromal cell-derived factor 2 like 1 (Sdf2l1) is required for normal glucose and lipid homeostasis^9^. Suppression of hepatic Sdf2l1 expression results in insulin resistance and hepatic steatosis with sustained ER stress in obese and diabetic mice and humans^9^. Thus, insufficient expression of hepatic FGF21 and Sdf2l1 may lead to insulin resistance and hepatosteatosis in obesity and type 2 diabetes.

On the other hand, serotonin (5-HT) is an endocrine hormone primarily produced by the gut and peripheral organs via tryptophan hydroxylase 1 (Tph1), and plasma 5-HT levels and expression of hepatic serotonin receptor 2a (htr2a) are increased in obese mice fed a high-fat diet^10^. On the other hand, inhibition of gut-derived 5-HT synthesis or hepatic htr2a expression suppresses high-fat diet-induced hepatic steatosis without altered energy expenditure^10^. Increased expression of hepatic htr2a in diet-induced obesity can therefore contribute to hepatosteatosis.

Whey protein isolate is a milk protein obtained after precipitation of casein during cheese production. Prolonged intake of whey protein isolate improves obesity, hepatosteatosis, insulin sensitivity, and glucose tolerance in mice fed a high-fat diet^11–14^, as well as in humans^15–17^. The effects of whey protein isolate on the altered expression of hepatic genes involved in hepatosteatosis, insulin resistance, glucose homeostasis, and the peripheral 5-HT system, however, remain unclear.

In the present study, we hypothesized that increases in hepatic FGF21 expression and circulating FGF21 levels precede diet-induced insulin resistance and hyperglycemia, which may be related to the increased expression of hepatic htr2a and Sdf2l1. Moreover, by suppressing peripheral 5-HT synthesis, intake of whey protein isolate may also suppress hepatic FGF21 production. To test our hypothesis, we first examined the changes in hepatic FGF21, Sdf2l1, htr2a expression, and plasma FGF21 levels in relation to body weight, blood glucose levels, and plasma insulin levels in mice fed a high-fat diet compared with a chow diet for 13 days. Second, we examined the effect of whey protein isolate intake on these changes in the mice. Third, we examined the effect of whey protein isolate intake on plasma 5-HT levels and the role of peripheral 5-HT in the regulation of hepatic FGF21 production in mice. Finally, we examined the relationship between htr2a and Sdf2l1 expression in the liver using a selective htr2a agonist in mice.

## Results

**Figure 1:**
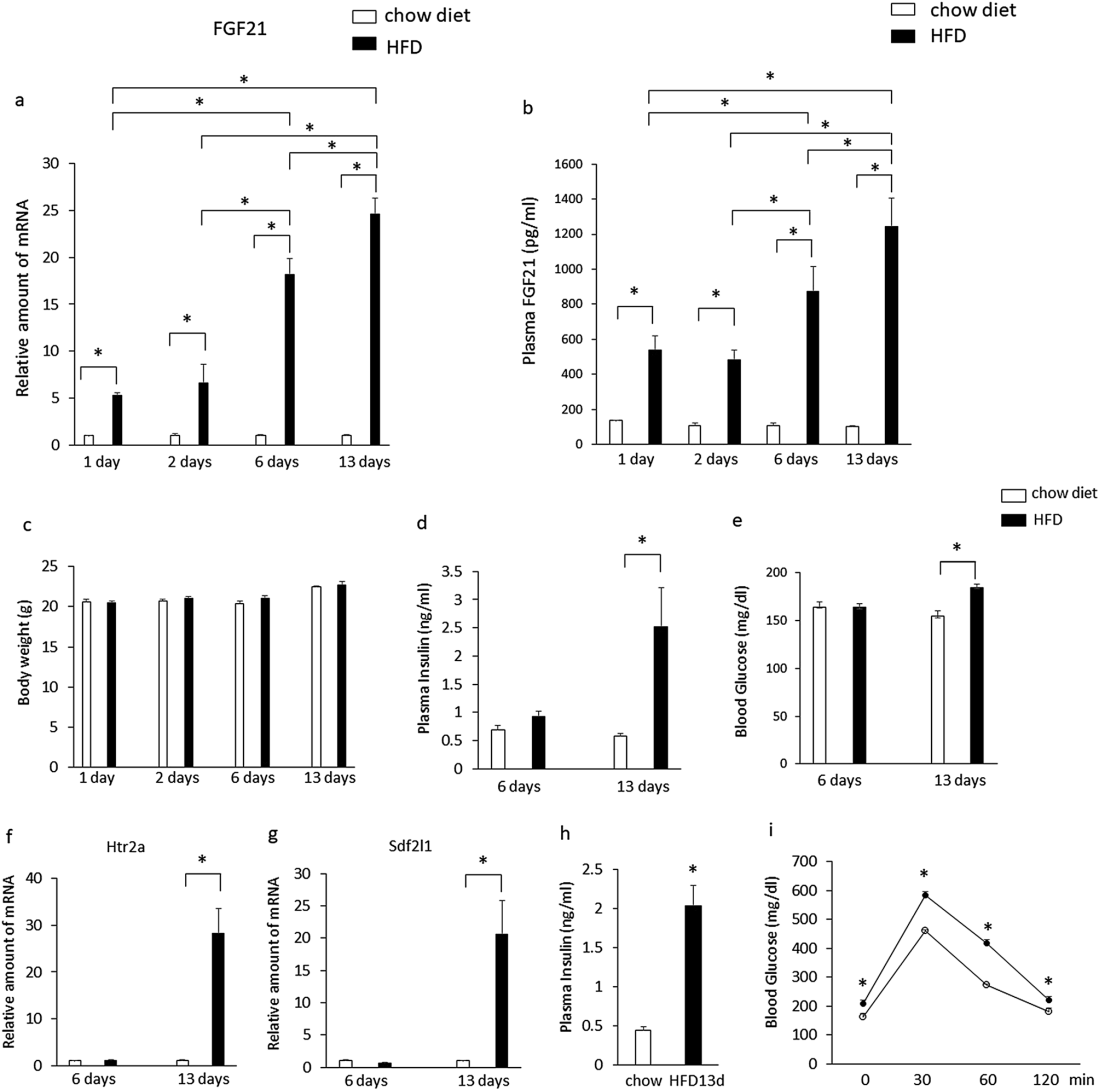
Expression of hepatic FGF21 (**a**), plasma FGF21 levels (**b**), body weight (**c**), plasma insulin (**d**) and blood glucose levels (**e**), expression of hepatic htr2a (**f**) and Sdf2l1 (**g**) in C57BL6J mice fed a high-fat diet (HFD) or a chow diet for 13 days. The relative amount of mRNA is shown as fold-change of the mean value of the control group in mice fed a chow diet (**a**,**g**,**h**). Plasma insulin levels were measured after a 6-h fast (**h**). Glucose tolerance was tested by injection of 1 g/kg d-glucose (**i**). Open symbols, mice fed a chow diet for 13 days; filled symbols, mice fed a high-fat diet for 13 days. Data are presented as the mean ± SEM (n = 6/group). *P < 0.05.

Expression of hepatic FGF21 (Fig. 1a) and plasma FGF21 levels (Fig. 1b) significantly increased in mice fed a high-fat diet for 1 day compared with a chow diet and gradually increased further over 13 days. Body weight did not differ significantly between mice fed a high-fat diet and a chow diet for 13 days (Fig. 1c). Plasma insulin levels (Fig. 1d), blood glucose levels (Fig. 1e), and expression of hepatic htr2a (Fig. 1f) and Sdf2l1 (Fig. 1g) were significantly increased in mice fed a high-fat diet for 13 days compared with mice fed a chow diet, whereas these measures did not differ significantly between mice fed a high-fat diet and a chow diet for 6 days. After a 6-h fast, mutant mice fed a high-fat diet for 13 days had both hyperinsulinemia (Fig. 1h) and elevated blood glucose levels (Fig. 1i), indicative of insulin resistance. The blood glucose levels were significantly elevated in mice fed a high-diet for 13 days compared with mice fed a chow diet at 30, 60, or 120 min after glucose administration, indicating impaired glucose tolerance (Fig. 1i). These findings suggest that increases in plasma FGF21 levels and expression of hepatic FGF21 precede hyperinsulinemia, hyperglycemia, and body weight gain in mice fed a high-fat diet, and that increased expression of hepatic htr2a and Sdf2l1 is associated with insulin resistance in mice fed a high-fat diet.

**Figure 2:**
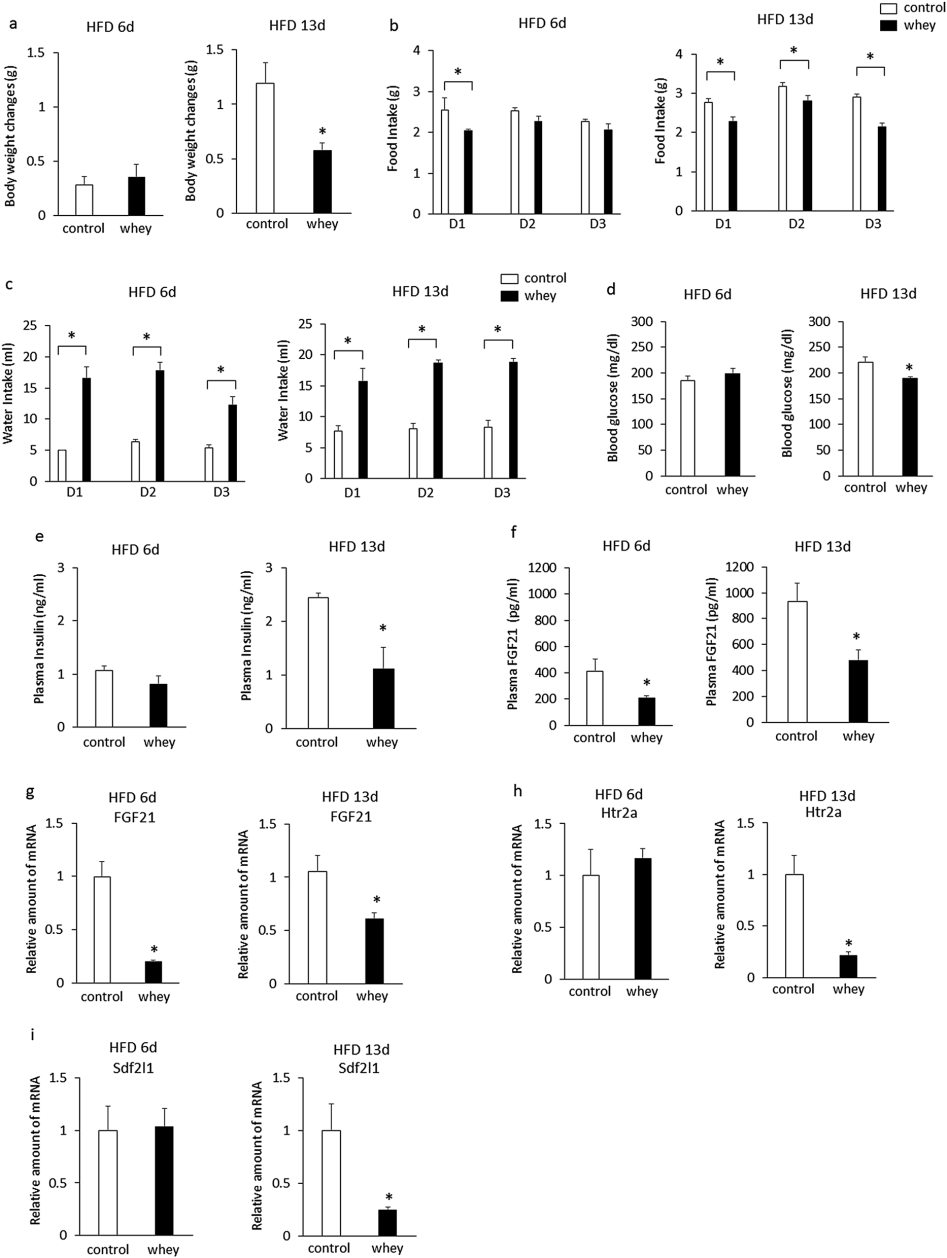
Effects of intake of whey protein isolate (5 g/100 ml water) on body weight changes (**a**), daily food intake (**b**), water intake (**c**), blood glucose levels (**d**), plasma insulin (**e**) and FGF21 (**f**) levels, expression of hepatic FGF21 (**g**), htr2a (**h**), and Sdf2l1 (**i**) in C57BL6J mice fed a high-fat diet for 6 days and 13 days. Body weights in mice fed a high-fat diet for 6 days were 21.3 g ± 0.3 g (controls) and 21.5 ± 0.2 g (whey group), respectively. Body weights in mice fed a high-fat diet for 13 days were 22.6 g ± 0.3 g (controls) and 22.3 ± 0.2 g (whey group), respectively. The relative amount of mRNA is shown as fold-change of the mean value of the control group in mice fed a high-fat diet (**g**,**h**,**i**). Data are presented as the mean ± SEM (n = 6/group). * P < 0.05. HFD; high-fat diet.

Ingestion of whey protein isolate for 3 days had no effect on body weight gain in mice fed a high-fat diet for 6 days, but significantly decreased body weight gain in mice fed a high-fat diet for 13 days compared with controls (Fig. 2a). Ingestion of whey protein isolate significantly decreased daily food intake on day 1 in mice fed a high-fat diet for 6 days, but significantly decreased daily food intake for 3 days in mice fed a high-fat diet for 13 days compared with controls (Fig. 2b). Ingestion of whey protein isolate significantly increased daily water intake in mice fed a high-fat diet for 6 days or 13 days compared with controls (Fig. 2c). Ingestion of whey protein isolate did not affect blood glucose levels (Fig. 2d) or plasma insulin levels (Fig. 2e) in mice fed a high-fat diet for 6 days, but significantly decreased blood glucose levels (Fig. 2d) and plasma insulin levels (Fig. 2e) in mice fed a high-fat diet for 13 days compared with controls. Ingestion of whey protein isolate significantly decreased plasma FGF21 levels (Fig. 2f) and hepatic FGF21 expression (Fig. 2g) in mice fed a high-fat diet for 6 days or 13 days compared with controls. Ingestion of whey protein isolate did not affect the expression of hepatic htr2a (Fig. 2h) or Sdf2l1 (Fig. 2i) in mice fed a high-fat diet for 6 days, but significantly decreased the expression of hepatic htr2a (Fig. 2h) and Sdf2l1 (Fig. 2i) in mice fed a high-fat diet for 13 days compared with controls. The daily intake of whey protein was 0.825 ± 0.09 g on day 1, 0.887 ± 0.07 g on day 2, and 0.612 ± 0.06 g on day 3 in mice fed a high-fat diet for 6 days. The daily intake of whey protein was 0.78 ± 0.1 g on day 1, 0.93 ± 0.02 g on day 2, and 0.94 ± 0.02 g on day 3 in mice fed a high-fat diet for 13 days. These findings suggest that ingestion of whey protein isolate suppresses increases in plasma FGF21 levels and expression of hepatic FGF21 in mice fed a high fat-diet for 6 or 13 days, and that ingestion of whey protein isolate suppresses hyperinsulinemia, hyperglycemia, and the increased expression of hepatic htr2a and Sdf2l1 in mice fed a high-fat diet for 13 days, whereas it has no effects on plasma insulin levels, blood glucose levels, or the expression of hepatic htr2a and Sdf2l1 in mice fed a high-fat diet for 6 days.

**Figure 3:**
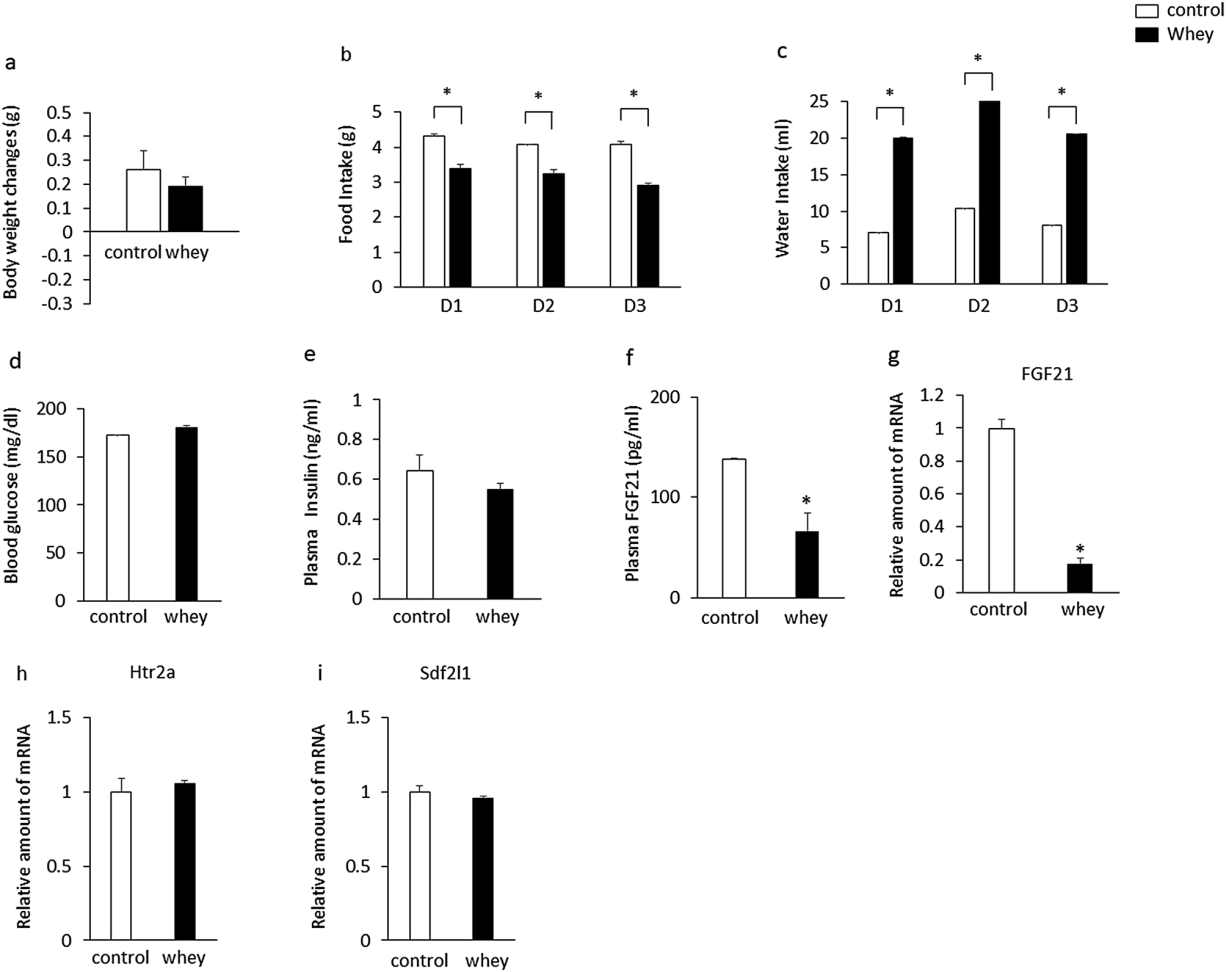
Effects of intake of whey protein isolate (5 g/100 ml water) on body weight changes (**a**), daily food intake (**b**), water intake (**c**), blood glucose levels (**d**), plasma insulin (**e**) and FGF21 (**f**) levels, expression of hepatic FGF21 (**g**), htr2a (**h**), and Sdf2l1 (**i**) in C57BL6J mice fed a chow diet for 13 days. Body weights in mice fed a chow diet for 3 days were 19.9 g ± 0.1 g (controls) and 19.8 ± 0.1 g (whey group), respectively. The relative amount of mRNA is shown as fold-change of the mean value of the control group in mice fed a chow diet (**g**,**h**,**i**). Data are presented as the mean ± SEM (n = 6/group). *P < 0.05.

On the other hand, ingestion of whey protein isolate for 3 days had no significant effect on body weight (Fig. 3a), but significantly decreased daily food intake (Fig. 3b) in mice fed a chow diet for 13 days. Daily water intake (Fig. 3c) was significantly increased in mice ingesting whey protein isolate compared with controls. Ingestion of whey protein isolate had no significant effect on blood glucose levels (Fig. 3d) or plasma insulin levels (Fig. 3e), but significantly decreased plasma FGF21 levels (Fig. 3f) and the expression of hepatic FGF21 (Fig. 3g) in mice fed a chow diet for 13 days compared with controls. Ingestion of whey protein isolate had no significant effect on the expression of hepatic htr2a (Fig. 3h) and Sdf2l1 (Fig. 3i) in mice fed a chow diet for 13 days. The daily intake of whey protein was 1.0 ± 0 g on day 1, 1.28 ± 0.03 g on day 2, and 1.03 ± 0.02 g on day 3. These findings suggest that ingestion of whey protein isolate downregulates plasma FGF21 levels and expression of hepatic FGF21 independently of the expression of hepatic htr2a and Sdf2l1, body weight, and plasma insulin and blood glucose levels in mice fed a chow diet.

**Figure 4:**
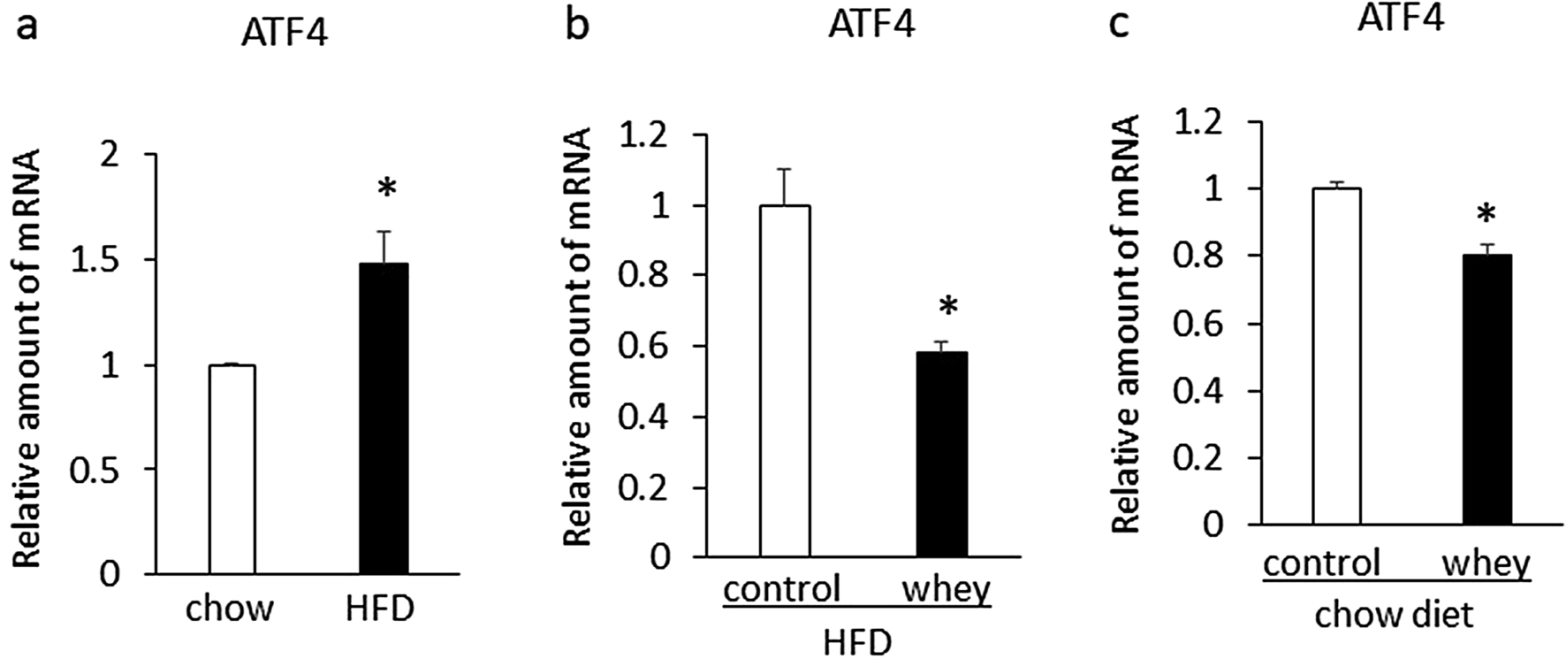
Expression of hepatic ATF4 in C57BL6J mice fed a high-fat diet or chow fat diet for 13 Days (**a**). Effects of intake of whey protein isolate (5 g/100 ml water) on expression of hepatic ATF4 in C57BL6J mice fed a high fat diet (**b**) or a chow diet (**c**) for 13 days. The relative amount of mRNA is shown as fold-change of the mean value of the control group in mice fed a chow diet. Data are presented as the mean ± SEM (n = 6/group). *P < 0.05.

Expression of hepatic activating transcription factor 4 (ATF4), which is a transcriptional factor of FGF21, was significantly increased in mice fed a high-fat diet for 13 days compared with mice fed a chow diet (Fig. 4a). Ingestion of whey protein isolate for 3 days significantly suppressed the increased expression of hepatic ATF4 compared with controls in mice fed a high-fat diet for 13 days (Fig. 4b). Moreover, ingestion of whey protein isolate for 3 days significantly suppressed expression of hepatic ATF4 compared with controls in mice fed a chow diet for 13 days (Fig. 4c). Although expression of hepatic PPARα was significantly increased in mice fed a high-fat diet for 13 days compared with mice fed a chow diet, ingestion of whey protein isolate for 3 days had no significant effect on the increased expression of hepatic PPARα compared with controls in mice fed a high-fat diet (data not shown). These findings suggest that ingestion of whey protein isolate downregulates expression of hepatic ATF4 in mice fed either a high-fat diet or a chow diet.

**Figure 5:**
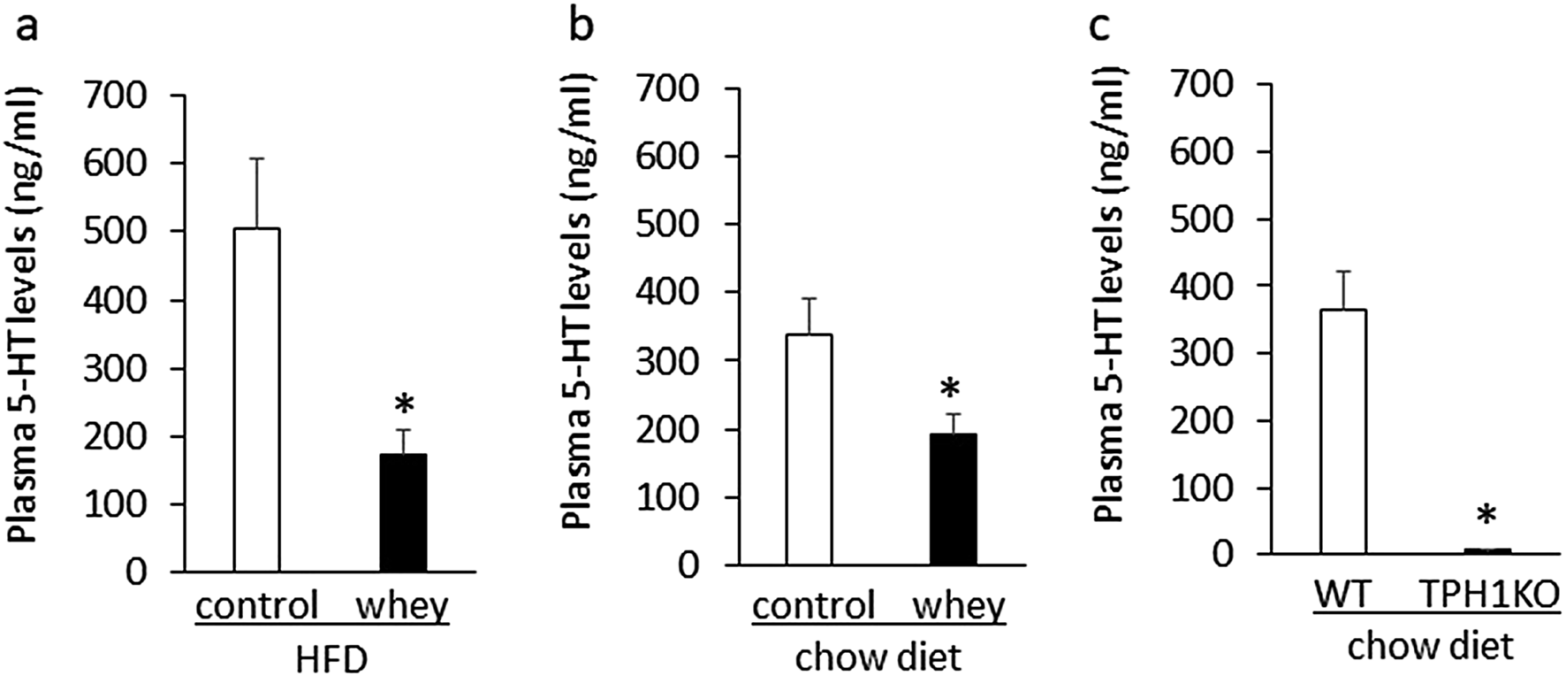
Effects of intake of whey protein isolate (5 g/100 ml water) on plasma 5-HT levels in mice fed a high-fat diet for 13 days (**a**) or a chow diet (**b**) for 13 days. Plasma 5-HT levels in 8-week-old Tph1 mutant mice and wild-type mice fed a chow diet (**c**). HFD; high-fat diet, Tph1KO; Tph1 mutant mice WT; wild-type mice.

To determine the effect of whey protein isolate on peripheral 5-HT synthesis, we examined the effects of whey protein isolate on plasma 5-HT levels in mice fed a high-fa diet or a chow diet for 13 days. Ingestion of whey protein isolate for 3 days significantly decreased plasma 5-HT levels in mice fed either a high-fat diet for 13 days (Fig. 5a) or a chow diet for 13 days (Fig. 5b) compared with controls. In addition, plasma 5-HT levels were very low in 8-week-old Tph1 mutant mice fed a chow diet compared with age-matched wild-type mice (Fig. 5c). These findings suggest that intake of whey protein isolate suppresses plasma 5-HT levels in mice fed either a high-fat diet or a chow diet.

**Figure 6:**
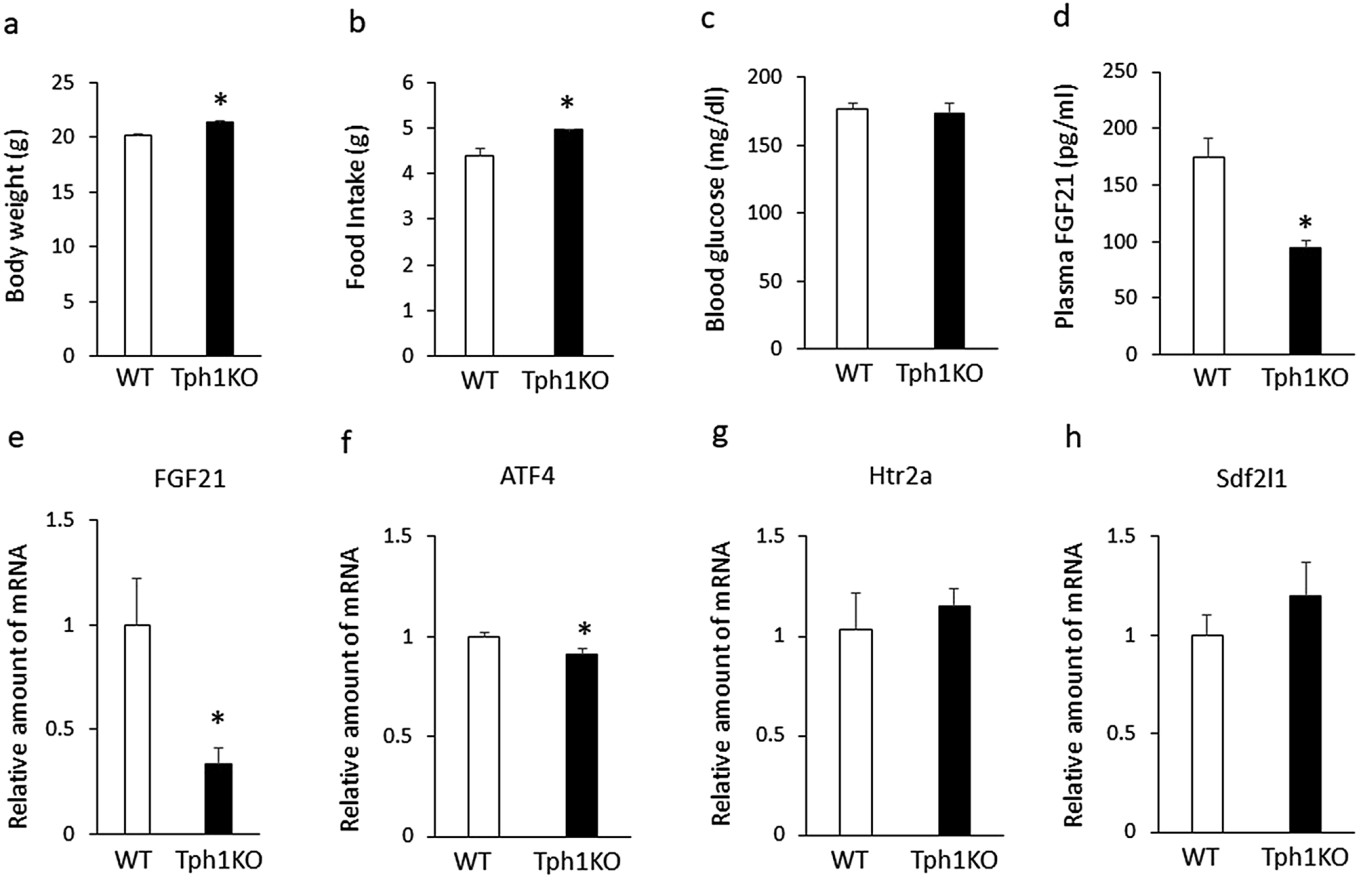
Body weight (**a**), daily food intake (**b**), blood glucose levels (**c**), plasma FGF21 levels (**d**) and expression of hepatic FGF21 (**e**), ATF4 (**f**), Htr2a (g), and Sdf2l1 (**h**) in Tph1 mutant mice (Tph1KO) and wild-type mice (WT) fed a chow fat diet. The relative amount of mRNA is shown as fold-change of the mean value of the WT group in mice fed a chow diet. Data are presented as the mean ± SEM (n = 6/group). * P < 0.05.

To further determine role of peripheral 5-HT in hepatic FGF21 production, we examined effect of genetic ablation of Tph1 on hepatic FGF21 expression in mice fed a chow diet. Although body weight (Fig. 6a) and daily food intake (Fig. 6b) were significantly increased in 8-week-old Tph1 mutant mice compared with age-matched wild-type mice, there were no significant differences in blood glucose levels between Tph1 mutant mice and wild-type mice (Fig. 6c). Plasma FGF21 levels (Fig. 6d) and expression of hepatic FGF21 (Fig. 6e) and ATF4 (Fig. 6f) were significantly decreased in Tph1 mutant mice compared with age-matched wild-type mice. There were no significant differences in hepatic htr2a (Fig. 6g) and Sdf2l1 (Fig. 6h) expression between Tph1 mutant mice and wild-type mice. These findings suggest that genetic ablation of Tph1 decreases hepatic FGF21 expression and plasma FGF21 levels in mice.

**Figure 7:**
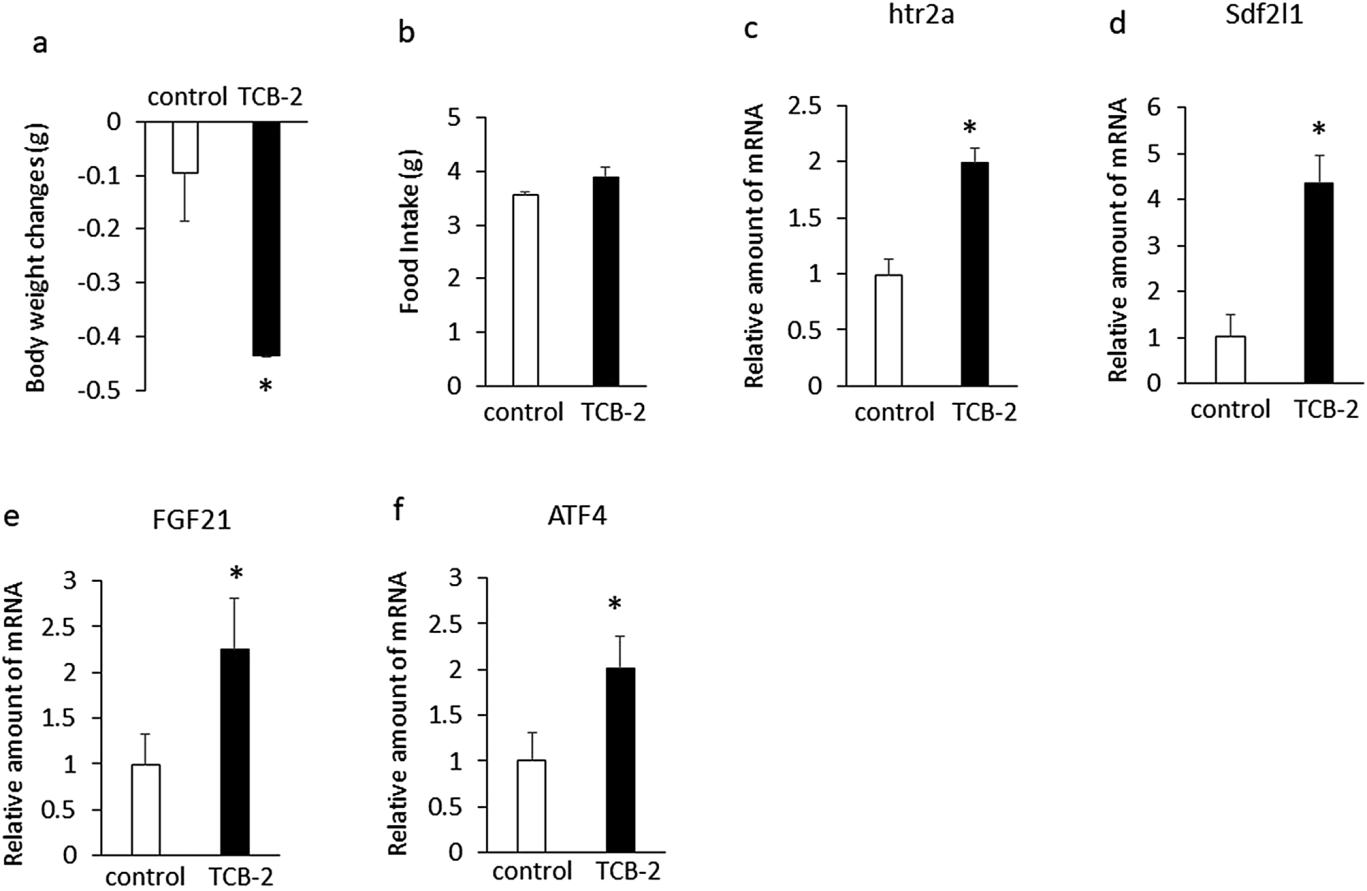
Effects of intraperitoneal injection of TCB-2 (2.5 mg/kg) or saline on body weight changes (**a**), daily food intake (**b**), and expression of hepatic htr2a (**c**), Sdf2l1 (**d**), FGF21 (**e**), and ATF4 (**f**) in C57BL6J mice fed a chow diet. Body weights in mice fed a chow diet for 3 days were 19.0 g ± 0.9 g (controls) and 19.8 ± 0.7 g (TCB-2 group), respectively. The relative amount of mRNA is shown as fold-change of the mean value of the control group in mice fed a chow diet. Data are presented as the mean ± SEM (n = 6/group). *P < 0.05.

Moreover, to determine the relationship between htr2a and Sdf2l1 expression in the liver, we examined the effect of pharmacologic stimulation of hepatic htr2a expression on the expression of hepatic Sdf2l1 in mice fed a chow diet. Treatment with TCB2, a high affinity htr2a agonist, significantly decreased body weight compared with controls (Fig. 7a), whereas it did not affect daily food intake (Fig. 7b) in mice fed a chow diet for 13 days. Treatment with TCB-2 increased expression of hepatic htr2a (Fig. 7c) compared with controls in mice fed a chow diet. In addition, treatment with TCB-2 significantly increased expression of Sdf2l1 (Fig. 7d), FGF21 (Fig. 7e) and ATF4 (Fig. 7f) compared with controls in mice fed a chow diet. These findings suggest that htr2a gene upregulates Sdf2l1 and FGF21 expression in the liver.

## Discussion

Increased circulating FGF21 levels are suggested to reflect a compensatory response to metabolic disturbances in obesity and/or type 2 diabetes^1^, and “FGF21 resistance” is suggested to be a pathophysiological mechanism of obesity^18^. On the other hand, prospective cohort studies demonstrated that FGF21 resistance precedes the onset of metabolic syndrome and type 2 diabetes in humans, suggesting that higher plasma FGF21 levels are an independent predictor of metabolic syndrome and type 2 diabetes^19,20^. The results of the present study support the findings of prospective cohort studies, demonstrating that plasma FGF21 levels and expression of hepatic FGF21 were acutely increased following intake of a high fat-diet for 1 day, and that the increases in plasma FGF21 levels preceded hyperinsulinemia, hyperglycemia, and weight gain in mice fed a high-fat diet. Thus, increases in circulating FGF21 levels do not result from obesity, and chronic increases in plasma FGF21 levels may precede diet-induced metabolic disturbances.

Mice with liver-specific FGF21 knockout fed a high-fat diet exhibit enhanced insulin resistance^1^. Our results, however, demonstrated that whey protein isolate-induced suppression of hepatic FGF21 leads to suppression of insulin resistance and hyperglycemia in mice fed a high-fat diet. The discrepancy could be due to the differences in genetic ablation of liver-derived FGF21 and a nutrient-induced normalization of circulating FGF21 levels. Not only depleted circulating FGF21, but also higher circulating FGF21 in mice fed a high-fat diet might contribute to insulin resistance.

Suppression of hepatic Sdf2l1 expression reportedly results in insulin resistance with sustained ER stress in obese and diabetic db/db mice with leptin receptor mutation^9^. Our results, however, demonstrated that hepatic Sdf2l1 expression was increased in insulin-resistant mice fed a high-fat diet, and that the suppression of increased hepatic Sdf2l1 expression induced by whey protein isolate improved insulin resistance in mice fed a high-fat diet. The discrepancy might be due to the differences in db/db mice and C57BL6J mice fed a high-fat diet. Whereas C57BL6J mice fed a high-fat diet for 13 days exhibit mild hyperglycemia and normal body weight, db/db mice exhibit remarkable hyperglycemia and obesity. In addition, whereas C57BL6J mice fed a high-fat diet show increased hepatic FGF21 expression and plasma FGF21 levels, db/db mice show decreased hepatic FGF21 expression and plasma FGF21 levels^21^. On the basis of these findings, altered expression of hepatic FGF21 might be related to altered expression of hepatic Sdf2l1.

Expression of hepatic htr2a is increased in obese mice fed a high-fat diet for 8 weeks^10^. Increased gut-derived 5-HT signals via htr2a in the liver contribute to hepatic steatosis in mice fed a high fat diet for 8 weeks^10^. Our results demonstrated remarkable increases in the expression of both hepatic htr2a and Sdf2l1 in insulin-resistant mice fed a high fat-diet for 13 days, although neither hepatic htr2a nor Sdf2l1 expression was increased in non-insulin resistant mice. These findings suggest that the increased expression of hepatic htr2a and Sdf2l1 occurs before weight gain and is related to insulin resistance in mice fed a high-fat diet. In addition, our findings indicate that the htr2a gene might upregulate Sdf2l1 expression in the liver. Moreover, htr2a might also upregulate FGF21 expression in the liver. The increased expression of hepatic htr2a might further enhance the increased expression of FGF21 in insulin-resistant mice fed a high fat-diet. Thus, the increased expression of hepatic Sdf2l1 and FGF21 via htr2a might be related to insulin resistance in mice fed a high-fat diet.

Although protein intake may modulate FGF21 synthesis, changes in FGF21 synthesis may depend on the type of protein ingested. Intake of soy protein β-conglycinin activates ATF4, leading to increased hepatic FGF21 expression and plasma FGF21 levels compared with intake of casein, a milk protein, in mice fed a chow diet^22^. In contrast to soy protein β-conglycinin, intake of whey protein isolate decreased expression of hepatic ATF4 and FGF21 in mice fed either a chow diet or a high-fat diet. Thus, whey protein isolate and soy protein β-conglycinin have different effects on FGF21 synthesis. Whey protein isolate-induced suppression of hepatic FGF21 production is not always due to increased protein intake.

Inhibition of peripheral 5-HT synthesis decreases body weight gain via increased thermogenesis, leading to improvement of insulin resistance and glucose homeostasis in mice fed a high-fat diet^23^. Our results demonstrated that intake of whey protein isolate decreased plasma 5-HT levels in mice. Intake of whey protein isolate may therefore suppress peripheral 5-HT synthesis, leading to the improvement of both insulin resistance and hyperglycemia in mice fed a high-fat diet. In addition, our results suggest that peripheral 5-HT is essential to maintain hepatic FGF21 expression and plasma FGF21 levels in mice. Whey protein isolate may therefore suppress peripheral 5-HT synthesis, leading to decreased hepatic FGF21 production. The whey protein isolate-induced normalization of hepatic FGF21 production may contribute to the improvement of hyperinsulinemia and hyperglycemia in mice fed a high-fat diet. Despite decreases in peripheral 5-HT synthesis and hepatic FGF21 production, whey protein isolate and genetic ablation of Tph1 had different effects on food intake in mice. On the basis of these findings, the decrease in circulating 5-HT may contribute to the inhibitory effect of the whey protein isolate on hepatic FGF21 production independently of food intake.

In summary, these findings suggest that increases in plasma FGF21 levels and hepatic FGF21 expression precede diet-induced weight gain, hyperinsulinemia, and hyperglycemia, and that intake of whey protein isolate could inhibit hepatic FGF21 production by suppressing peripheral 5-HT synthesis in mice.

## Methods

### General procedures

Male C57BL6J mice (5 weeks old) were purchased from Japan CLEA. The mice were individually housed in cages with free access to water and chow pellets in a light- and temperature-controlled environment (12 h on/12 h off, lights on at 08:00; 20–22 °C).

In the first experiment, 5-week-old C57BL6J mice fed a high-fat diet (High Fat Diet 32, 60% calories mainly from fat: Japan CLEA) or a chow diet (Labo MR Stock, Nosan Co, Japan) for 1 day, 2 days, 6 days, or 13 days were decapitated, and blood was obtained for the measurement of plasma FGF21 and insulin levels. The liver was dissected for determining mRNA levels.

In the second experiment, 5-week-old C57BL6J mice were fed a high-fat diet for 6 days with or without whey protein isolate (5 g/100 ml water) for 3 days (days 3 through 6). Daily water intake and food intake and body weight changes were determined. On day 6, the animals were decapitated and blood was obtained for the measurement of blood glucose, plasma FGF21 and insulin levels. The liver was dissected out for determining mRNA levels.

In the third experiment, 5-week-old C57BL6J mice were fed a high-fat diet for 13 days with or without whey protein isolate (5 g/100 ml water) for 3 days (days 10 through 13). Daily water intake and food intake and body weight changes were determined. On day 13, the animals were decapitated and blood was obtained for the measurement of blood glucose, plasma FGF21, insulin, and 5-HT levels. The liver was dissected out for determining mRNA levels.

In the fourth experiment, 5-week-old C57BL6J mice were fed a chow diet for 13 days with or without whey protein isolate (5 g/100 ml water) for 3 days (days 10 through 13). Daily water intake and food intake and body weight changes were determined. On day 13, the animals were decapitated and blood was obtained for the measurement of blood glucose, plasma FGF21, insulin, and 5-HT levels. The liver was dissected for determining mRNA levels.

In the fifth experiment, body weight and daily food intake were determined in 8-week-old Tph1 mutant mice and wild-type mice fed a chow diet. The animals were decapitated and blood was obtained for the measurement of blood glucose, plasma FGF21, and 5-HT levels. The liver was dissected out for determining mRNA levels.

Finally, 5-week-old C57BL6J mice were fed a chow diet for 13 days. The mice were intraperitoneally injected with saline or the high-affinity htr2a agonist, 4-Bromo-3,6-dimethoxybenzocyclobuten-1-yl methylamine hydrobromide (TCB-2) (2.5 mg/kg), twice daily for a day. Body weight changes and daily food intake were determined. 24 h later, the liver was dissected out for determining mRNA levels.

The experiments were performed between 14:00–16:00. Whey protein isolate (Provon 190; protein 93%, water 3.5%, lipid 0.4%, and ash 2.8%; pH 6.0–6.5) was obtained from Glanbia Nutritionals (Niseikyoeki Co, Japan). The dose of TCB-2 (2.5 mg/kg) was selected based on evidence that TCB-2 had no significant effect on food intake^24^.

### Glucose homeostasis

After a 6-h fast (08:30 to 14:30), plasma insulin and glucose levels were measured, and glucose tolerance was tested following intraperitoneal injection of 1 g/kg d-glucose. Blood samples from the tail vein were taken at 0, 15, 30, 60 and 120 min and blood glucose levels were measured as described previously^25^.

### Tph1 mutant mice

Homozygous mutant males bearing a null mutation of the Tph1 gene (congenic on a C57BL/6 N background) and age-matched wild-type mice were used. The line has been maintained through mating of females heterozygous for the Tph1 gene with heterozygous males obtained from Cyagen Biosciences Inc. Genomic DNA was extracted from tails of littermates using TaKaRa MiniBEST Universal Genomic DNA Extraction kit (Ver.5.0_Code No.9765). Genotypes were confirmed by PCR-LabChip (PerkinElmer LabChip GX Touch HT) analysis using the forward primer F1: 5′-ACATCAGCCTTCTGCTCTGTTTC-3′ and the reverse primer R1: 5′-TCACTGAGAGCATCAAGCCCAG-3′ and R2: 5′-ATTTCCGGGACTCGATGTGTAAC-3′. Tph1 mutant and wild-type alleles correspond to the 611- and 489-bp fragments, respectively.

Before the experiment, animals were all housed (3–5 mice per cage) with free access to water and chow pellets in a light—(12 h on/12 h off; lights off at 2000 h) and temperature—(20–22 °C) controlled environment. The animal studies were conducted in accordance with the institutional guidelines for animal experiments at Tohoku University Graduate School of Medicine and all experimental protocols were approved by the institutional committee at Tohoku University.

### Blood chemistry

Whole blood was mixed with EDTA-2Na (2 mg/ml) and aprotinin (500 kIU/ml) to determine the plasma levels of FGF21 and insulin. Plasma levels of FGF21 and insulin were measured by enzyme-linked immunosorbent assay (rat/mouse FGF21 ELISA Kit, R&D Systems, Tokyo, Japan; and a mouse Insulin ELISA Kit [TMB], AKRIN-011 T, Shibayagi, Gunma, Japan, respectively) as described previously^21,26,27^. Plasma 5-HT levels were measured by enzyme-linked immunosorbent assay (mouse 5-HT; BA E-5900, Labor Diagnostika Nord, Nordhorn, Germany). Blood glucose levels were measured using glucose strips (blood glucose monitoring system; Accu-Check, Roche Diagnostics, Tokyo, Japan).

### Real-time quantitative reverse transcription–polymerase chain reaction (RT–PCR)

Total RNA was isolated from mouse liver using the RNeasy Midi kit (Qiagen, Hilden, Germany) according to the manufacturer’s instructions. cDNA synthesis was performed using a Super Script III First-Strand Synthesis System for RT-PCR Kit (Invitrogen, Rockville, MD) with 1 μg total RNA. cDNA synthesized from total RNA was evaluated in a real-time PCR quantitative system (LightCycler Nano Instrument Roche Diagnostics, Mannheim, Germany). The primers were listed in supplementary Table 1.

The relative amount of mRNA was calculated using β-actin mRNA as the invariant control. Data are shown as fold-change of the mean value of the control group as described previously^21,26,27^.

### Statistical methods

Data are presented as mean ± SEM (n = 6). Comparisons between two groups were performed using Student’s t-test. A P value of less than 0.05 was considered statistically significant.

## Supplementary information

Supplementary Information 1.
