# Supplementary material for: Whey protein isolate inhibits hepatic FGF21 production, which precedes weight gain, hyperinsulinemia and hyperglycemia in mice fed a high-fat diet
Source: Sci Rep. 2020 Sep 25;10:15784. doi: 10.1038/s41598-020-72975-8 (PMC7519058; doi:10.1038/s41598-020-72975-8)
Supplement: Supplementary file 1 — Supplementary Information 1. [file 41598_2020_72975_MOESM1_ESM.docx]

Whey protein isolate inhibits hepatic FGF21 production, which precedes weight gain, hyperinsulinemia and hyperglycemia in mice fed a high-fat diet.

Katsunori Nonogaki, Takao Kaji

**Supplementary information**

**Supplementary Table 1.**

| **GENE** |  | **SEQUENCE** |
| --- | --- | --- |
| **FGF21** | sense | CACCGCAGTCCAGAAAGTC |
|  | antisense | ATCAAAGTGAGGCGATCCA |
| **Sdf2l1** | sense | CACACGGTCCAATAGCAGTG |
|  | antisense | GCTCTAGACCTCTGCGCTTC |
| **Htr2a** | sense | TTCAGTGCCAGTACAAGGAG |
|  | antisense | GAGTGTTGGTTCCCTAGTGTAA |
| **ATF4** | sense | AGACACCGGCAAGGAGGATG |
|  | antisense | CGAAACAGAGCATCGAAGTCAAC |
| **β-actin** | sense | TTGTAACCAACTGGGACGATATGG |
|  | antisense | GATCTTGATCTTCATGGTGCTAGG |
